# Prevalence of Anxiety and Depression in Patients With Pulmonary Hypertension and Their Impact on Health-Related Quality of Life: A Prospective Cross-Sectional Observational Study

**DOI:** 10.7759/cureus.109819

**Published:** 2026-05-28

**Authors:** Sarfraz Saleemi, Reem Alameer, Toka Alsulaim, Naif Alghasab, Sulaman AlMesned

**Affiliations:** 1 Department of Internal Medicine, King Faisal Specialist Hospital and Research Center, Riyadh, SAU; 2 Department of Medicine, King Faisal Specialist Hospital and Research Center, Riyadh, SAU; 3 Department of Internal Medicine, Medical College, Hail University, Hail, SAU; 4 Department of Emergency, Critical Care and Anesthesia, Medical College, Qassim University, Qassim, SAU

**Keywords:** anxiety, depression, hospital anxiety depression scale (hads), pulmonary hypertension, quality of life

## Abstract

Introduction

Pulmonary hypertension (PH) is a chronic progressive disease that affects quality of life (QoL). The prevalence of mood disorders in PH patients in Saudi Arabia is not known. The aim of this study was to investigate the prevalence of anxiety and depression in patients with PH and to examine their association with health-related quality of life (HRQoL).

Methods

This was a prospective cross-sectional observational study. In this study, 42 PH patients were evaluated for mood disorders using the Hospital Anxiety and Depression Scale (HADS), and HRQoL was assessed using the Short Form 36 (SF-36) Health Survey.

Results

In the current study, 42 PH patients were evaluated. The cohort's mean age was 42.8±13.3 years, and 31 (74%) were women. Mean pulmonary artery pressure was 46.4±15 mm Hg, and 25 (60%) patients had severe PH (mean pulmonary artery pressure (mPAP)>45 mmHg). Thirty-eight (90%) patients were in World Health Organization Functional Class (WHO-FC) II/III, and four (10%) were in WHO-FC IV. HADS anxiety and depression scores were elevated (11-21) in four (10%) and six (14%) patients, respectively. The presence of anxiety and/or depression affected HRQoL.

Conclusion

Anxiety and depression were infrequent among PH patients. Further studies with formal psychosocial evaluation are needed to confirm these findings.

## Introduction

Pulmonary hypertension (PH) is a chronic progressive disease that carries high mortality and morbidity [1]. Dyspnea on exertion due to elevated pulmonary artery pressure, increased pulmonary vascular resistance, and right heart failure is the most common symptom that limits daily physical activity [2]. The quality of life of patients with PH is affected by many aspects, such as limitations on physical activity that may lead to social isolation and unemployment. As a result, the patients are at risk of developing mental health problems such as depression and anxiety [3,4].

Mental disorders such as depression and anxiety are common in PH patients. Löwe et al. reported that depression was related to the degree of symptoms and functional limitation. The prevalence of major depression increased from 7.7% in patients with New York Heart Association functional class (FC) I to 45% in FC-IV [5]. McCollister et al. looked into the prevalence of depression in patients with PH in the outpatient setting. The result of the study showed that up to 55% of PH patients seen in two PH referral centers in the United States suffered from depressive symptoms, and 15% had major depressive disorder [6]. Another study found that the prevalence of depression and anxiety in PH patients was 53% and 51%, respectively [7].

The largest PH registry in the United States, called the REVEAL Registry (Registry to Evaluate Early and Long-term PAH Disease Management), has shown that 25% of PH patients have depression compared to 6.7% in the general population [2]. Other mood disorders are also common in these patients. An international survey of PH patients revealed feelings of worthlessness (22%), frustration (35%), anger (24%), and reduced pleasure in activities (25%) compared with before their PH diagnosis [4].

King Faisal Specialist Hospital and Research Center has a dedicated PH program. The prevalence of depression and anxiety in patients suffering from PH in the Arab world has not been well characterized, where family and cultural traditions are different from those in the Western world. This provides a chance to study the prevalence of depression and anxiety in this group of patients and to look into their impact on the quality of life.

The objectives of this study were to estimate the prevalence of moderate-to-severe anxiety and depression among patients with pulmonary hypertension using the Hospital Anxiety and Depression Scale (HADS), to examine their association with health-related quality of life across Short Form 36 (SF-36) domains, and to explore their relationship with disease severity as reflected by functional class and hemodynamic parameters.

This study presents region-specific data on psychological distress among patients with pulmonary hypertension, situated within a sociocultural context that has been inadequately represented in prior research. By integrating standardized psychological assessments with clinical measures of disease severity and quality of life, the study enhances our understanding of potential variations in the psychosocial burden of pulmonary hypertension when compared to previously reported international cohorts.

A preprint version of this manuscript was previously posted on the medRxiv preprint server on April 22, 2021.

## Materials and methods

Study design

This prospective, cross-sectional, observational study was conducted at King Faisal Specialist Hospital and Research Centre, Riyadh, and was approved by the ethical board of the centre (ORA/0561/37). Participants were patients >14 years of age with a confirmed diagnosis of PH by right heart catheterization, defined as mean pulmonary artery pressure (mPAP) >25 mmHg, pulmonary capillary wedge pressure (PCWP) <15, and pulmonary vascular resistance (PVR) >3 Wood units [8].

PH was categorized into mild, moderate, and severe based on mPAP (25-34, 35-44, >45 mmHg, respectively) [9]. The patients were on PH-specific treatment for at least three months and had regular follow-up at the PH clinic. Patients aged <14 years, pregnant ladies, and patients with known mental disorders before the diagnosis of PH were excluded. Specific questionnaires were used to assess for depression, anxiety, and health-related quality of life.

Data were collected from the electronic medical record, including demographics, clinical characteristics, PH class, hemodynamic parameters, and treatment details. Informed consent was obtained in view of the prospective nature of the study, and it did not involve any therapeutic intervention.

The sample size was established based on the number of consecutive eligible patients diagnosed with confirmed PH who attended the specialized PH clinic during the study period. Due to the exploratory nature of the research and the limited population size associated with this rare condition, a formal a priori sample size calculation was not feasible. A post-hoc power analysis indicated that a sample of 42 participants would provide approximately 80% power to detect moderate effect sizes (Cohen's d≈0.8) in comparisons of HRQoL scores across HADS severity groups at a two-sided alpha level of 0.05.

Assessment of depression, anxiety, and health-related quality of life

In the current study, the following tools were used and were free to use.

HADS, developed in 1983 by Zigmond and Snaith, was used for screening [10]. The HADS is a 14-item scale; seven of the items relate to anxiety and seven to depression. Validity of the HADS was studied by Ingvar Bjelland et al. and was found to be a good tool in assessing the symptom severity of anxiety disorders and depression in both somatic, psychiatric, and primary care patients and in the general population [11]. The Arabic version of the HADS scale has been validated in several studies [12]. HADS scores range from 0 to 21. A score of 0-7 is considered normal, 8-10 mild or possible, 11-14 moderate or probable, and 15-21 severe or definite [13]. Patients in the category of moderate to severe scores were considered to have a mental disorder, anxiety, and/or depression. Licence was obtained from Mapi Research Trust to use the Arabic version of HADS.

The Short Form 36 (SF-36) Health Survey was used to assess the quality of life in patients with PH. Health-related quality of life (HRQoL) is a person’s perceived quality of life, reflecting satisfaction with the areas of life usually affected by a chronic illness. HRQoL scores can assess the impact of chronic disease on the patient’s quality of life and serve as an indicator of improvement after starting the appropriate management [14,15].

The SF-36 questionnaire was developed in the United States by the Boston Health Research Institute. It is mainly used to assess various aspects of the quality of life of adults. It became the most widely used method for estimating the health status of the general population because it is easy to use and interpret. The reliability and validity of the SF-36 questionnaire have been assessed in multiple studies worldwide [14,15]. It involves eight scaled scores, which are the averages of the specific questions for each scale. Each scale is graded from 0 to 100; the higher the score, the less disability, and vice versa.

Statistical analysis

Data were analyzed using SPSS version 22 (IBM Corp, Armonk, NY). Continuous variables are presented as mean±standard deviation, and categorical variables are presented as frequencies and percentages. Comparisons between HADS categories (normal/mild vs. moderate/severe) were performed using independent-samples t-tests. P values <0.05 were considered statistically significant.

## Results

Forty-two patients were included in the study, with a mean age of 42.8±13.3 years, and 31 (74%) were women. Moreover, 19 (45%) patients were married, 18 (43%) were financially independent, and 14 (33%) had post-secondary education. The mean pulmonary artery pressure (mPAP) of the cohort was 46.4±15.1 mmHg. Twenty-five (60%) patients had severe PH, 12 (28%) moderate, and five (12%) mild PH (mPAP >45, 35-44, and 25-34 mmHg, respectively). Seventeen (40%) patients were on single pulmonary vasodilator treatment, while the rest were on combination therapy. Twelve (30%) patients had comorbid conditions, including diabetes mellitus, hypertension, hypothyroidism, and chronic kidney disease. Thirty-eight (90%) patients had World Health Organization Functional Class (WHO-FC) II/III, and four had class IV condition (Table 1).

**Table 1:** Baseline demographic and clinical characteristics and HADS anxiety and depression scores of patients with pulmonary hypertension [8,10]. PH: Pulmonary Hypertension.  HADS: Hospital Anxiety and Depression Scale

Characteristics	Value
Age, mean±SD	42.8±13.3
Females, n (%)	31 (74%)
WHO Functional Class, n (%)	
II/III	38 (90%)
IV	4 (10%)
PH Group, n (%)	
Group 1	29 (69%)
Group 4	9 (21%)
Other	4 (10%)
Severity of PH, n (%)	
Mild	5 (12%)
Moderate	12 (28%)
Severe	25 (60%)
HADS Anxiety score, mean±SD	
Overall, n=42	5.4±4
Normal to mild (0-10), n=38	4.5±2.7
Moderate to severe (10-21), n=4	14.0±2.9
HADS Depression score, mean±SD	
Overall, n=42	4.9±6
Normal to mild (0-10), n=36	3.3±2.7
Moderate to severe (10-21), n=6	13.8±2.8

Moreover, none of the patients reported substance abuse. The mean HADS anxiety and depression scores overall were 5.4±4 and 4.9±6, respectively. HADS anxiety score was 0-10 (normal-mild) in 38 (90%) patients, and four (10%) patients had a score of 11-21 (moderate-severe). Thirty-six patients (86%) had HADS depression scores of 0-10 and six (14%) had a score of 11-21 (Table 1).

**Figure 1:**
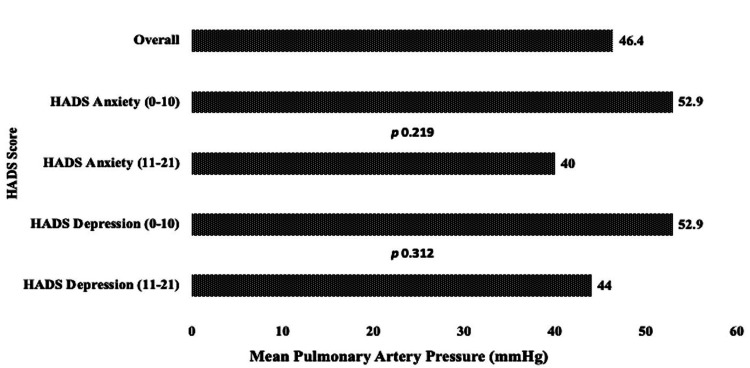
Relationship between HADS score and mean pulmonary artery pressure. Hospital Anxiety and Depression Scale (HADS) [10].

There was no significant difference in the severity of PH between normal-mild and moderate-severe HADS score group (p=0.219 for anxiety and p=0.312 for depression). Figure 1 shows similar mPAP values across HADS severity groups, with no linear association observed between mPAP and HADS anxiety or depression scores.

**Figure 2:**
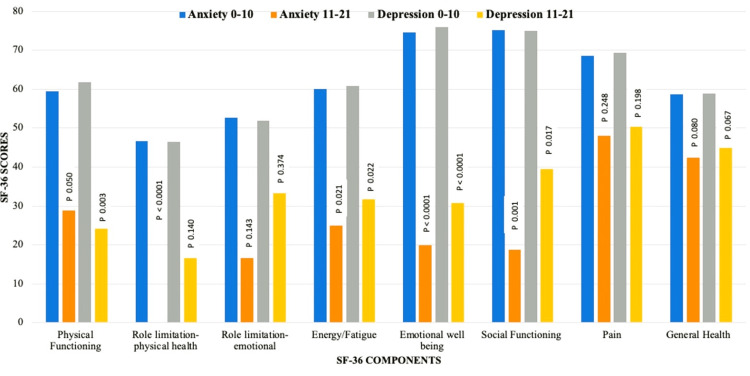
Comparison of SF-36 scores between normal-mild and moderate-severe HADS groups Hospital Anxiety and Depression Scale (HADS) [10] and Short Form 36 (SF-36) Health Survey [14].

The presence of anxiety and/or depression affected five components in the SF-36 Quality of Life assessment, namely physical functioning, physical role performance, vitality, emotional well-being, and social functioning (p<0.05). There was no significant difference in bodily pain, general health perception, and emotional role performance scores (Table 2 and Figure 2).

**Table 2:** Pulmonary hemodynamics and SF-36 scores according to HADS anxiety and depression severity [10,14]. Values are mean±SD. Continuous variables were compared using Welch independent-samples t-tests. mPAP: Mean pulmonary artery pressure.

Variable	Anxiety HADS 0-10 (n=38)	Anxiety HADS 11-21 (n=4)	t value	P value	Depression HADS 0-10 (n=36)	Depression HADS 11-21 (n=6)	t value	P value
Pulmonary hemodynamics								
Right atrial pressure	11.4±5.5	15.5±4.5	−1.69	0.165	11.9±5.8	11.5±3.7	0.22	0.828
Cardiac index	2.5±0.8	2.69±0.66	−0.54	0.621	2.5±0.8	2.6±0.4	−0.47	0.643
mPAP	52.9±20.3	40.0±10.0	2.15	0.074	52.9±20.8	44.0±9.4	1.72	0.106
Quality of Life (SF-36)								
Physical functioning	59.5±30.1	28.8±8.5	4.74	<0.001	61.9±27.8	24.2±23.1	3.59	0.008
Role limitation – physical	46.7±45.8	0±0	6.29	<0.0001	46.5±45.6	16.7±40.8	1.63	0.146
Role limitation - emotional	52.7±47.5	16.6±19.1	2.94	0.019	51.9±48.8	33.3±29.9	1.27	0.233
Energy/fatigue	60.0±28.4	25.0±17.8	3.49	0.019	60.8±28.7	31.7±19.7	3.11	0.013
Emotional well-being	74.7±23.8	20.0±17.6	5.69	0.004	75.9±22.4	30.7±8.9	8.68	<0.0001
Social functioning	75.3±30.8	18.8±23.9	4.36	0.011	75.0±32.7	39.6±30.0	2.64	0.033
Pain	68.7±32.1	48.1±43.8	0.92	0.421	69.4±32.1	50.4±38.5	1.14	0.295
General health	58.8±17.7	42.5±11.9	2.47	0.062	59.0±18.1	45.0±30.4	1.10	0.318

## Discussion

This prospective observational study shows the prevalence of anxiety and depression in PH patients in this particular region. The low prevalence of mood disorders (10% anxiety and 14% depression) in this cohort is very different from the published studies, where the prevalence is significantly higher.

PH is a chronic, progressive, and debilitating disease. The presence of exertional dyspnea and fatigue, the most common symptoms, affects daily activities and quality of life. PH poses a significant clinical challenge, marked by symptoms like dyspnea and fatigue that limit physical capacity and contribute to deconditioning and psychological distress [16].

The presence of anxiety and/or depression may further deteriorate the already compromised quality of life. The reported prevalence of anxiety and depression in patients with PH is high, and the HADS score has been shown to correlate with HRQoL [17,18].

The findings of the current study diverge markedly from global benchmarks. According to a Swiss study, 51% anxiety and 53% depression in 91 Swiss PH patients at baseline, with partial remission post-therapy [7]. Another study documented 28% anxiety/depression disorders in 117 German PH cases, independent of PH severity [18]. Even Asian subgroups in Mai et al.'s (2022) meta-analysis showed higher rates (~40%), suggesting regional variance beyond ethnicity [19].

Importantly, cultural stigma associated with mental illness and the underreporting of psychological symptoms in conservative societies may partially elucidate the lower observed rates, as previously documented in Middle Eastern populations [20]. This discrepancy challenges universal diathesis-stress models by Monroe and Simons, which posit that PH, as a stressor, should uniformly exacerbate vulnerability, and highlights the moderating role of sociocultural factors [21].

In a previous study, patients diagnosed with pulmonary arterial hypertension and chronic thromboembolic PH indicated that more than two-thirds of subjects met the criteria for anxiety and/or depression. Furthermore, these psychological symptoms were significantly associated with lower SF-36 scores across multiple quality-of-life domains [22].

The psychological impact of PH may be explained by a biopsychosocial framework in which disease burden interacts with psychological and social factors. Sociocultural influences such as family support and stigma may therefore contribute to regional differences in the prevalence of anxiety and depression. Previous studies indicate that psychological distress correlates more strongly with subjective symptoms such as dyspnea and functional limitation than with objective hemodynamic parameters, highlighting the complex biopsychosocial interaction underlying disease burden. Predictive models have further demonstrated that depression, anxiety, stress, and social support collectively impact health-related quality of life. Accordingly, the relatively low prevalence of moderate-severe mood disorders observed in our cohort may partially reflect differences in social support; however, the absence of formal psychosocial assessment precludes definitive conclusions [23].

This difference can be explained by the social, cultural, and family setup in Saudi Arabia, where joint family living and close social contact and support play an important role in alleviating the patient’s mental suffering. The fact that the presence of mood disorders in this study does not show any correlation to the severity of PH suggests that other factors, such as social isolation, unemployment, and lack of financial support may cause these disorders. The role of family and social support may help to prevent these disorders and improve the quality of life [24].

Nevertheless, this interpretation remains provisional, as family support, socioeconomic status, and stigma-related reporting bias were not directly assessed in this study. Previous research has indicated that perceived social support significantly moderates the severity of depression in individuals with chronic cardiopulmonary disease, highlighting the necessity for structured psychosocial assessments in future investigations [23,24]. The relationship between anxiety and PH is characterized by a complex biopsychosocial feedback loop. While our data showed no statistically significant difference in mPAP between patients with and without moderate-to-severe anxiety (p=0.219), the clinical "worsening" of the disease was evident in the subjective domain. Anxiety and depression appear to act as exacerbating factors for physical symptoms; for instance, patients in the high-anxiety group reported physical functioning scores that were more than 50% lower than those of their less-anxious counterparts.

Furthermore, the limited sample size of patients exhibiting moderate-to-severe anxiety (n=4) and depression (n=6) significantly restricts statistical power and heightens the risk of Type I error, particularly in light of multiple unadjusted comparisons across the SF-36 domains. Consequently, these findings should be interpreted with caution and regarded as hypothesis-generating rather than confirmatory.

Limitations

This study has several limitations. First, the small sample size, particularly within the moderate-to-severe HADS groups, limits statistical power; post hoc considerations suggest adequate power only for detecting moderate-to-large effects, and therefore the findings should be interpreted as exploratory and hypothesis-generating. Second, the single-center design may restrict generalizability. Third, measurement limitations exist, as anxiety and depression were assessed using the HADS, a screening rather than diagnostic tool, psychosocial factors were not directly measured, and multiple comparisons were performed without adjustment. Finally, cultural reporting bias may have influenced results due to potential underreporting related to stigma, absence of psychiatric diagnostic confirmation, lack of a control group, and reliance on self-reported measures. Larger multicenter studies incorporating structured psychiatric assessments and formal evaluation of social support are needed to confirm these findings.

## Conclusions

This study highlights the presence of mood disorders in PH patients in this region. The prevalence of anxiety and depression in PH patients in this cohort is low. Close family ties, cultural differences, and organized social services may explain the low prevalence. PH is linked to psychological distress that can negatively impact patients' quality of life. Within this cohort, the prevalence of anxiety and depression was observed to be lower than that reported in numerous international studies; however, the presence of mood disorders was correlated with significant impairment across various HRQoL domains.

These findings indicate that the psychological burden associated with PH may not be comprehensively captured by disease severity or hemodynamic parameters alone. Sociocultural and psychosocial factors may shape the experience and reporting of psychological symptoms, underscoring the need for contextual considerations when interpreting prevalence estimates. Routine psychological screening may still be warranted, as mood disorders considerably compromise health-related quality of life, even when their prevalence appears low. Integrating mental health assessments into standard pulmonary hypertension care may promote the early identification of psychological distress and enhance comprehensive patient management. Future multicenter studies employing larger sample sizes and structured psychiatric evaluations are essential to more accurately delineate the relationship between psychological health, sociocultural influences, and clinical outcomes in pulmonary hypertension.
